# Conditional generative adversarial networks applied to EEG data can inform about the inter-relation of antagonistic behaviors on a neural level

**DOI:** 10.1038/s42003-022-03091-8

**Published:** 2022-02-21

**Authors:** Amirali Vahid, Moritz Mückschel, Sebastian Stober, Ann-Kathrin Stock, Christian Beste

**Affiliations:** 1 Cognitive Neurophysiology, Department of Child and Adolescent Psychiatry, Faculty of Medicine, TU Dresden, Dresden, Deutschland; 2 Artificial Intelligence Lab, Institute for Intelligent Cooperating Systems, Faculty of Computer Science, Otto von Guericke University Magdeburg, Magdeburg, Germany

**Keywords:** Cognitive neuroscience, Human behaviour

## Abstract

Goal-directed actions frequently require a balance between antagonistic processes (e.g., executing and inhibiting a response), often showing an interdependency concerning what constitutes goal-directed behavior. While an inter-dependency of antagonistic actions is well described at a behavioral level, a possible inter-dependency of underlying processes at a neuronal level is still enigmatic. However, if there is an interdependency, it should be possible to predict the neurophysiological processes underlying inhibitory control based on the neural processes underlying speeded automatic responses. Based on that rationale, we applied artificial intelligence and source localization methods to human EEG recordings from N = 255 participants undergoing a response inhibition experiment (Go/Nogo task). We show that the amplitude and timing of scalp potentials and their functional neuroanatomical sources during inhibitory control can be inferred by conditional generative adversarial networks (cGANs) using neurophysiological data recorded during response execution. We provide insights into possible limitations in the use of cGANs to delineate the interdependency of antagonistic actions on a neurophysiological level. Nevertheless, artificial intelligence methods can provide information about interdependencies between opposing cognitive processes on a neurophysiological level with relevance for cognitive theory.

## Abstract (web summary)

An artificial intelligence algorithm trained on EEG recordings can be used to predict brain dynamics underpinning motor inhibition processes using neurophysiological information from motor execution.

## Introduction

Goal-directed behavior is central to coping with everyday life demands. In many situations, we are confronted with trade-offs between partly antagonistic adaptive constraints^1,2^, and goal-directed behavior often requires a balance or arbitration between antagonistic behaviors, termed “metacontrol” in current theoretical frameworks^2–4^. For example, automated responding can be advantageous on the one hand because it is fast and requires few cognitive resources to be carried out^5^. On the other hand, automated response tendencies are difficult to control or inhibit^6^, which is disadvantageous once that behavior becomes inappropriate and needs to be inhibited^7^. This illustrates that antagonistic processes show an interdependency concerning what constitutes goal-directed behavior, as suggested by metacontrol theoretical frameworks. Several lines of evidence support this, e.g., showing that speeded habitual responding to environmental demands makes the inhibition of these responses more error-prone^7–11^. In Go/Nogo experiments, which allow assessing this nexus, two categories of trials are presented: Go trials, which require a speeded response to a stimulus, and Nogo trials, which require the inhibition of that speeded response. Whenever Go trials occur with high frequency, participants tend to establish automated (speeded) response tendencies that are particularly difficult to inhibit^8–11^. When assuming that this interdependency is also reflected on the neural level, it should be possible to predict the neural processes underlying inhibitory control based on the neural processes underlying speeded automatic responses. However, such insights into the nature of this neural-level interdependency have lacked until now. Many existing studies have analyzed the inter-relation or differences between response execution (Go) and inhibition trials (Nogo) in terms of their scalp topography and electroencephalography (EEG) electrode site-specific effects^12–14^ and also functional imaging studies often examine the difference (contrast) between Go and Nogo trials^15^. While an inter-relation of Go and Nogo trials is thus inherent to the data analysis strategy in many existing studies examining response inhibition the fundamental question of whether Go trial neurophysiological signal properties can predict Nogo trial neurophysiological activity is quite elusive. Specifically, it is unknown whether knowledge about the time course of neurophysiological processes underlying one form of goal-directed behavior (e.g., speeded responses execution) provides sufficient information to infer/predict the time course and neurophysiological pattern of processes of an antagonistic form of goal-directed behavior (e.g., response inhibition) has not been answered.

The most likely reasons for this are that neurophysiological data is noisy and that there are likely complex non-linear interdependencies in the time course of the neural correlates^16^ of cognitive processes involved in goal-directed behavior^17,18^. Using only some aspects (parameters) or selected time points from the neurophysiological data (e.g., amplitude information at specific time points as it is the case in classical event-related potential research) would be too simplistic, as such an approach would not allow accounting for the time course of the neurophysiological activity underlying goal-directed behavior. Deep learning models are powerful tools to estimate the mapping between two groups of multi-dimensional, non-linearly related variables or time courses (i.e., X and Y). Unlike linear models such as linear regression, there is no assumption about an existing (linear) relationship between variables. By increasing the model’s capacity (i.e., number of layers and neurons) deep learning can estimate highly non-linear interrelations. EEG data are highly non-linear and non-stationary. Deep learning models are widely used in many images and signal recognition and synthesizing tasks. The distribution of the images and signals is very high dimensional, sparse, and among all features, there are significant correlations. These properties cause learning the probability distribution function (i.e., P data) is not possible. Therefore, we applied conditional generative adversarial networks (cGANs) to human EEG recordings from a Go/Nogo response inhibition experiment to provide insights into the interdependency of neural processes underlying antagonistic behavioral tendencies. A cGAN can generate high-quality data not depending on an estimate of Pdata directly. Instead, it estimates the ratio between Pdata and the Pmodel. We hypothesized that the application of cGANs allows generating the precise neurophysiological pattern and the entire time course of neural activity during inhibitory control (i.e., EEG signals during Nogo trials) based on neurophysiological data from speeded response trials (i.e., EEG signals during Go trials)—and vice versa. The generated neurophysiological pattern should then show high similarities with recorded data. If this was the case, applying artificial intelligence methods to EEG data could inform cognitive science and provide information about the principles underlying antagonistic classes of goal-directed behavior on a neurophysiological level. This would not only further our understanding of interdependencies between distinct cognitive processes on a neural level, but it may in the long-range also provide an opportunity to test the predictions of computational modeling, inform future theories, and—potentially—allow for the prediction of behavioral performance in various situations.

## Results

### Behavioral data

For descriptive data, the mean and the standard error of the mean are given. The mean percentage of correct responses in Go trials was 98.75% ± .07. The mean reaction time on Go trials was 348.76 ms ± 2.2. False alarms occurred in 11.86% ± 0.54 of all Nogo trials. The values were computed using IBM SPSS Statistics 27.

### EEG correlates of response inhibition

**Fig. 1:**
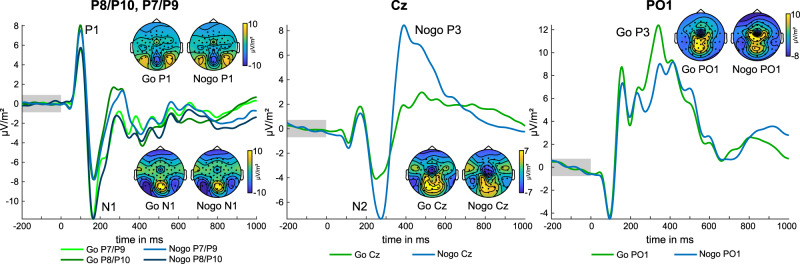
Event-related potentials (ERP) for Go and Nogo trials. P1 and N1 ERPs are depicted at electrodes P7/P9 and P8/P10. N2 ERP is depicted at electrode Cz, P3 ERP at electrode Cz, and PO1. Go trial EEG data are depicted in green color, Nogo data in blue color. Additionally, scalp topography plots show voltage distribution at the time point of the respective ERP peak. Timepoint zero denotes the time point of stimulus onset.

ERP components of Go and Nogo trials are shown in Fig. 1 for all electrodes.

For the P1 amplitudes at electrodes P7/P9 and P8/P10, a repeated-measures ANOVA with the factors “condition” and “electrodes” revealed a significant main effect of electrodes (*F*(1254) = 39.36; *p* < 0.001; *η*^2^_p_ = 0.134) showing that the P1 amplitude was smaller over the left hemisphere (5.35 µV/m^2^ ± 0.28) than over the right hemisphere (7.43 µV/m^2^ ± 0.32). The main effect of condition was not significant (*F*(1254) = 3.04; *p* = 0.083; *η*^2^_p_ = 0.012). Additionally, the interaction of condition × electrode yielded significance (*F*(1254) = 22.99; *p* < 0.001; *η*^2^_p_ = 0.083). As shown by Bonferroni-corrected post hoc *t*-tests, P1 amplitudes differed between Go (7.66 µV/m^2^ ± 0.32) and Nogo  trials (7.21 µV/m^2^ ± 0.32) over the right hemisphere (*t*(255) = 4.53; *p* < 0.001), but not over the left hemisphere (Go: 5.27 µV/m^2^ ± 0.28; Nogo: 5.42 µV/m^2^ ± 0.28; *t*(255) = −1.43; *p* > 0.31).

For the N1 amplitudes at electrodes P7/P9 and P8/P10, the ANOVA showed a main effect of electrode (*F*(1254) = 101.92; *p* < 0.001; *η*^2^_p_ = 0.286) indicating that the N1 amplitude was larger (i.e., more negative) over the left hemisphere (−11.11 µV/m^2^ ± 0.36) than over the right hemisphere (−7.05 µV/m^2^ ± 0.35). All other effects were not significant (*F* < 2.77; *p* > 0.096). The N2 amplitude at electrode Cz was significantly larger (i.e., more negative) in Nogo trials (−7.09 µV/m^2^ ± 0.39) than in Go trials (−3.9 µV/m^2^ ± 0.32), as indicated by a significant main effect of condition (*F*(1254) = 143.92; *p* < 0.001; *η*^2^_p_ = 0.362)) in the corresponding ANOVA. The frontocentral P3 amplitude at electrode FC1 was significantly larger (i.e., more positive) in Nogo trials (8.25 µV/m^2^ ± 0.54) than in Go trials (1.86 µV/m^2^ ± 0.38), as indicated by the ANOVA (*F*(1254) = 259.61; *p* < 0.001; *η*^2^_p_ = 0.505). The ANOVA for the parietal P3 amplitude at electrode P1 (parietal P3) also showed a significant main effect of condition (*F*(1254) = 19.71; *p* < 0.001; *η*^2^_p_ = 0.072), indicating that the parietal P3 amplitude was more positive in Go trials (9.26 µV/m^2^ ± 0.33) than in Nogo trials (8.32 µV/m^2^ ± 0.34).

### GANs generate EEG correlates of inhibition in early time windows

**Fig. 2:**
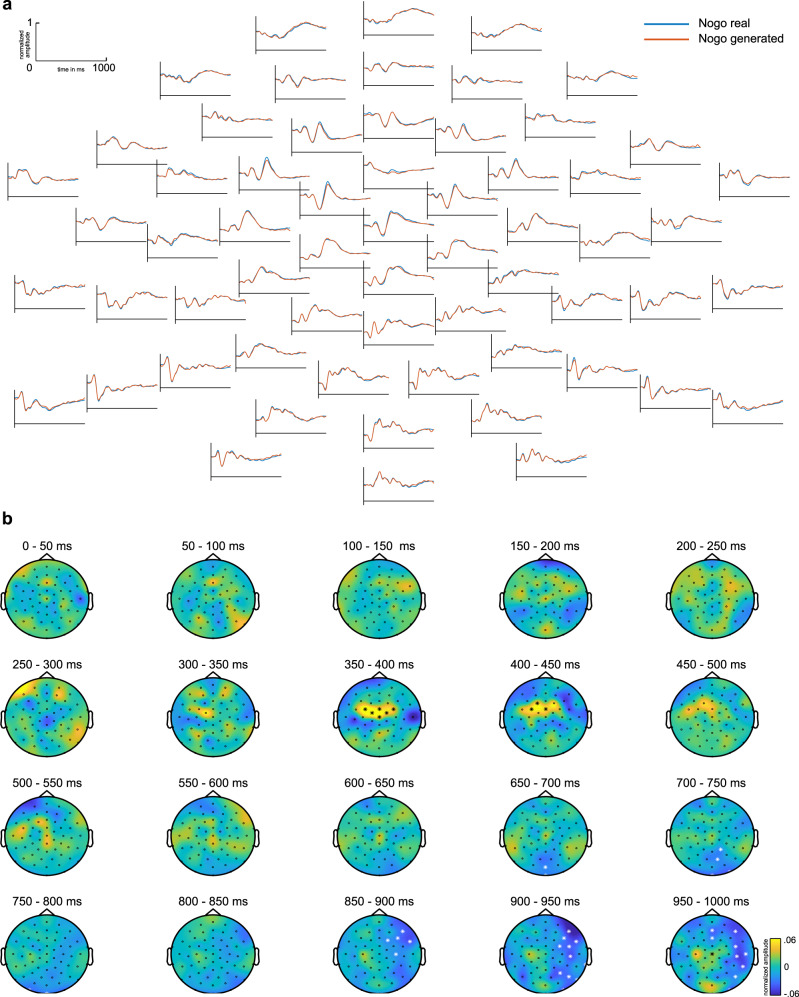
The generated Nogo signal at all electrodes. **a** Real (blue) and generated (red) Nogo trial EEG data as well as the difference of Real and generated Nogo EEG signal (purple). The *x*-axis denotes time in ms from stimulus onset, *y*-axis signal amplitude. **b** Results of cluster-based permutation analysis comparing real and generated Nogo signal, aggregated in 50 ms steps. Electrodes showing significant differences in the respective time step are marked by an asterisk, positive differences by a black, and negative differences by a white asterisk. Colors denote amplitude differences. The figures show normalized amplitudes.

After normalization of Go and Nogo EEG data, the Go data was fed into the cGAN in the time window from 0 to 1000 ms. Figure 2a shows the cGAN-generated Nogo signal based on the Go signal (i.e., the Nogo is conditioned on the Go signal) in comparison to the measured (real) Nogo signal for all electrodes.

**Fig. 3:**
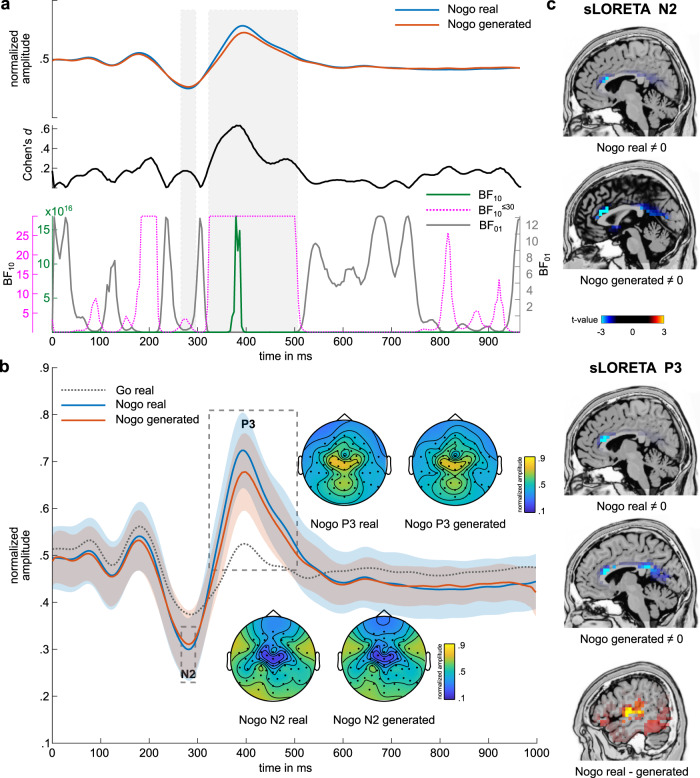
The generated Nogo signal at pooled electrodes Cz, FCz, FC1, FC2, FC3, and FC4. **a** Pooled and EEG signal for real Nogo and generated Nogo signal (top). Cohen’s effect sizes are based on a *t*-test comparing each sample point between generated and real Nogo signal (middle). Bayes factor bf01 (gray) and bf10 (green) for each sample point compared between generated and real Nogo signal. The dotted purple line depicts bf10 with a cut-off value of 30 (i.e., values above 30 were set to 30). Gray boxes indicate the time window of the N2 and the P3. **b** Real (blue) and generated (orange) Nogo signal as well as real Go signal (dotted gray) at pooled electrodes Cz, FCz, FC1, FC2, FC3, and FC4. The blue and orange shadings indicate the standard deviation. Timepoint zero denotes the time point of stimulus onset. Gray boxes indicate time windows of N2 and P3 analysis. Topography plots showing the voltage distribution are given for real and generated Nogo signals in the N2 peak time windows and in the P3 time window. Colors denote amplitude differences. **c** Results of sLORETA analysis. In the N2 time window, comparing the Nogo real signal against zero, sLORETA revealed sources in BA24. Comparing the generated Nogo signal against 0, sources in BA24 were found. For the P3 time window, comparing the real Nogo signal against 0 showed sources in BA24. Comparing the generated Nogo signal against 0, sources in BA24 and BA31 were revealed. Contrasting real and generated Nogo signal, sLORETA revealed sources in BA13. The figures show normalized amplitudes. For the sLORETA, *t*-values are given.

As can be seen, there is a high visual similarity between the cGAN-generated and the real Nogo signal in all channels. The time window from stimulus onset until response execution is of particular interest for this analysis because it encompasses traditionally investigated neurophysiological correlates of perceptual processing, attentional control, response selection, and motor response execution. Cluster-based permutation tests with 1000 random draws were used to compare the measured and the cGAN-generated Nogo signal. The test compared all electrodes at all time points from 0 to 1000 ms. The test results are shown in Fig. 2b, where they have been temporally aggregated in 50 ms steps. No significant clusters were obtained in the N2 time window (i.e., from 271 to 291 ms). As shown in Fig. 3a, b, by visual inspection, a close visual correspondence of generated and real Nogo signal in the time window of the N2 at frontocentral sites: The generated signal closely matches the real signal in shape as well as in amplitude. The permutation test showed no differences at a cluster coinciding with the N2 time window. Bayesian single-sample *t*-tests in that time window (for pooled electrodes Cz, FCz, FC1, FC2, FC3, FC4, as revealed by a significant cluster in the P3 time range, see further below) yielded a mean Bayes factor BF_10_ (i.e., evidence for H1 over H0) of 2.42 (±0.47; 0.2713–0.2913 ms), suggesting only anecdotal evidence for an amplitude difference, despite the large sample size of *N* = 255 participants. Additionally, there was only a nominal mean effect size of *d* = 0.17 (±0.01) for the difference between the generated and real signal. The close resemblance between the cGAN-generated and the measured Nogo signal in the N2 time window was further corroborated by the results of the sLORETA source localization (see Fig. 3c), where the sources (BA24) for the cGAN-generated Nogo signal are also similar to the sources found for the measured Nogo data.

### Differences between GAN-generated and measured data in late time windows

**Fig. 4:**
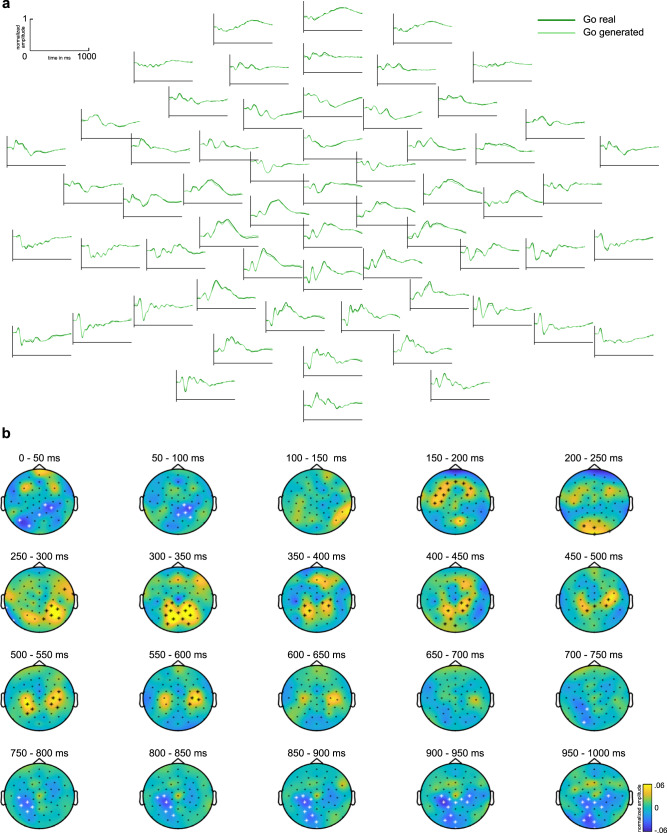
The generated Go signal. **a** Real (dark green) and generated (light green) Nogo trial EEG data as well as the difference of Real and generated Go EEG signal (blue). The *x*-axis denotes time in ms from stimulus onset, *y*-axis normalized signal amplitude. **b** Results of cluster-based permutation analysis comparing real and generated Go signal, aggregated in 50 ms steps. Electrodes showing significant differences in the respective time step are marked by an asterisk, positive differences by a black, and negative differences by a white asterisk. Colors denote amplitude differences. The figures show normalized amplitudes.

Opposed to earlier time windows reflecting inhibitory control sub-processes (i.e., the N2 time window), significant mid-central electrode clusters were obtained in the time window of the frontocentral P3 component (i.e., from 350 to 400 ms) at electrodes Cz, FCz, FC1, FC2, FC3, and FC4, as well as slightly smaller significant clusters from 400 to 450 ms (*p* < 0.013) (see Fig. 4a, b). After limiting the analysis to the mean activity of the time window from 350 to 400 ms, permutation tests confirmed that the mean amplitudes of the measured and cGAN-generated Nogo signal were significantly different in this time window (one positive cluster of mid-central electrodes; *p* < 0.001). Figure 3a presents a comparison of the measured (real) and cGAN-generated Nogo signal pooled over the electrodes contained in the significant clusters observed in the frontocentral P3 time window (i.e., Cz, FCz, FC1, FC2, FC3, and FC4). By visual comparison, the maximum amplitude of the cGAN-generated Nogo signal appears to be slightly smaller than the measured signal, whereas the overall shape of the waveform is quite similar. Bayesian *t*-tests conducted for each sample at the pooled electrodes revealed a mean BF_10_ > 100 (1.07 × 10^16^ ± 5.33 × 10^15^) in the time window from 324 to 504 ms overlapping with the P3 time window. A BF_10_ > 100 indicates robust evidence for amplitude differences between the real Nogo and the generated Nogo signal. However, the large sample size used here (*n* = 255) was necessary for cGANs and facilitated detecting differences that are very small and possibly only trivial and without biological meaning^19–21^. In this regard, it is essential that the observed amplitude differences showed only small to medium effect sizes (mean *d* = 0.40 ± 0.02). When conducting source localization analyses examining the source of the frontocentral P3 in the measured data and the cGAN-generated data, the two analyses revealed overlapping activations in the anterior cingulate cortex (BA24) (Fig. 3c). Thus, there are noteworthy similarities between the measured and the cGAN-generated data regarding the functional neuroanatomical sources despite the observed amplitude differences. Further contrasting the cGAN-generated and the measured Nogo data in the P3 time window revealed that amplitude differences in that time window were due to activation differences in the left anterior insular cortex (BA13) (Nogo_real_ > Nogo_generated_).

The cluster-based permutation tests also showed significant clusters from 650 to 750 ms and from 850 to 1000 ms after stimulus onset. Here, primarily parieto-occipital electrode sites were revealed. However, these differences occur after the response and are therefore outside of the crucial time window for response selection processes. The signal in the late post-response time windows is likely to exhibit high variability due to overlap with successive trials, which probably impairs the performance of the cGAN.

### GAN shows weaker performance generating Go data from Nogo data

Even though the generated Go signal visually resembles the real Go signal remarkably well, there seem to be more overall visual differences between the real and generated Go signal than between the real and generated Nogo signal (see Fig. 4a). This was corroborated by a cluster-based permutation test comparing the cGAN-generated and real Go signal, showing significant clusters (*p* < 0.05) in 17 out of 20-time epochs (50 ms) (see Fig. 4b). In contrast, significant clusters for comparing generated and real Nogo signals were observed only in 7 out of 20-time epochs. This shows that the cGAN performed better in generating Nogo trials from recorded Go trials than, conversely, in generating Go trials from recorded Nogo trials.

We applied the same cGAN architecture on a Simon task paradigm data published by Vahid et al.^18^, a sample consisting of *N* = 186 healthy adult participants. The results can be found in the supplemental material (Supplemental Fig. 1). These results show that the same cGAN architecture as applied for the Go/Nogo task was less well able to generate a signal in the Simon Task.

## Discussion

The goal of the current study was to examine whether artificial intelligence methods (i.e., a cGAN) can provide information about the interrelation of partly antagonistic behavioral tendencies on a neurophysiological level when applied to EEG data. It was not intended to develop and estimate the performance of a new GAN in comparison to other machine learning procedures. Instead, the study was motivated by the cognitive theory that complementary and antagonistic instances of goal-directed behavior are located on opposing ends of a continuum. Computational principles (parameters) may relate neurophysiological processes of partly antagonistic classes of goal-directed behavior^2–4^. Therefore, it was necessary to the information of one condition (i.e., Go or Nogo) as input for the cGAN and not an arbitrary or random signal as an input, which is done in conventional GANs. Using a random signal in a GAN, it is not possible to study the question of whether it is possible to predict Nogo trial neurophysiological activity using information from Go trials (and vice versa). For that reason, we chose to use EEG data from a well-defined experimental paradigm measuring clearly defined antagonistic behaviors as an example and input for the cGAN; i.e., we focused our analysis on the interplay between executing a speeded response and the ability to inhibit such a response using a Go/Nogo task. We used a well-established GAN architecture^22^ whose performance was already validated in various experiments^22^ with identical hyperparameters and network architecture as used in the current study. The modification changed all convolution and pooling layers from two dimensions into one dimension to be applicable for the EEG signal.

The results show that neural processes underlying partially antagonistic classes of goal-directed behavior can be related to each other. Time-resolved neurophysiological processes underlying one instance of goal-directed behavior (i.e., the speeded execution of a response) contain sufficient information to generate time-resolved neurophysiological processes of the antagonistic class of goal-directed behavior /cognitive process (i.e., response inhibition). In previous research, several lines of evidence have shown that response inhibition is associated with a ubiquitously occurring complex in the EEG N2 and P3 time window^14,23^. Notably, the response inhibition data generated based on real response execution data revealed strong similarities with real inhibition-related data in the N2 time window. This was further underlined by an analysis of the EEG sensor data as well as source reconstruction analyses. The latter showed highly similar activations for the generated and the real data in the anterior cingulate cortex. This is particularly noteworthy as sLORETA source localization depends on the input from all recorded electrodes. This evidences that the application of GAN methods did not only allow to generate remarkably similar data patterns at traditionally investigated electrodes sites but instead for all of the investigated electrodes.

However, still, we found amplitude differences between the real data and the GAN-generated data in the P3 time window. It has been suggested that the Nogo-N2 and Nogo-P3 components reflect different cognitive sub-processes involved in response inhibition^14^ also having partially different neurobiological correlates^24,25^. While neurophysiological modulations in the Nogo-N2 time window have been suggested to reflect conflict monitoring or pre-motor inhibition processes, modulations in the Nogo-P3 time window have been suggested to reflect the motor inhibition itself^14^. This suggests that the information contained in Go trials is limited. Not all cognitive sub-processes involved in response inhibition can be approximated equally well based on the neurophysiological dynamics exhibited during speeded responding. Interestingly, this would match the common interpretation that the Nogo-N2 reflects the (pre-)motor inhibition of an automated Go response tendency (i.e., directly relates to the Go process). In contrast, the Nogo-P3 either reflects motor inhibition itself or an evaluation thereof (i.e., might be more strongly related to the processes associated with inhibition than with the prepotent Go response tendency itself)^24,26,27^. Nevertheless, source localization analysis again revealed highly similar sources in the anterior cingulate cortex for the generated and the real Nogo data in the P3 time window. This suggests that the dynamics of neuronal activity can generally be mapped sufficiently well, even though the dynamics of speeded responses do not allow to precisely predict the strength of neuronal activation in response inhibition for every neurophysiological time point and hence for every response inhibition subprocess. This poses the question of which of the Nogo-P3-associated processes cannot be predicted based on Go trials? Contrasting the cGAN-generated and real Nogo amplitudes in the Nogo-P3 time window with source localization analysis revealed activation differences in the left anterior insular cortex (BA13) (Nogo_real_ > Nogo_generated_). Meta-analyses have shown the insular cortex to play an essential role in sensorimotor and cognitive processes^28,29^, especially in evaluative but also inhibitory processes during cognitive control^30–32^. Such evaluative aspects have also been suggested to be evident during response inhibition and are most likely reflected in the Nogo-P3 time window^14,24,27,33^, but this suggestion has also been questioned based on data from response interruption^34^. Still, it is well possible that the cGAN cannot capture such evaluative aspects coded in neurophysiological data because they neither arise nor directly relate to the processes of automated response tendencies reflected in the Go trial data. Another potential reason is that the experiment used a skewed distribution of Go and Nogo trials to induce a strong response tendency and thus to impose high demands on response inhibition processes^8–11^. The high frequency of Go trials makes responding somewhat automated^8–11^ and puts less emphasis on the execution of a controlled response. This lack of controlled/evaluative aspects in the Go trial neurophysiological signal likely makes it much harder, if not impossible, for the cGAN to generate neurophysiological data that reflects exactly these processes in Nogo trials and points towards the limitations of such methods in the context of cognitive neurophysiology. Considering this, it is also important to note that even though cGAN application allowed to generate a convincing time-resolved neurophysiological response inhibition signal based on neurophysiological response execution data, this was not equally possible the other way round. The most likely reason is that there were three times more Go trials than Nogo trials. Consequently, the Go data fed into the cGAN to generate a Nogo signal were less variable, which may have allowed for better cGAN performance. Yet, this skewed distribution of trials was necessary to induce a strong response tendency and impose high demands on response inhibition^8–11^. Since single-trial EEG data are noisier than averaged EEG data, we used the latter for the analyses performed. Future studies shall investigate and design deep learning models for the single-trial level.

An important future step is the optimization of the applied cGAN architecture. The cGAN applied here is inspired by a type of cGAN proposed by Isola et al.^22^, originally designed for the image to image translations. This architecture was optimized for example for translations of day-time pictures into night-time pictures or colorizing monochrome pictures. Here, predominantly color conversions of objects while maintaining the overall structure of the objects were required. To put the concept of changing the color without changing the objects into signal processing words, the model must learn how to change signal peaks/amplitudes without introducing latency for the new signal. The cGAN may therefore give satisfactory results when amplitude changes prevail, as is the case in a Go/Nogo experiment, but it can perform worse when significant latency shifts appear. This is suggested by the results shown in the supplemental material (Supplemental Fig. 1) using the same cGAN architecture for a Simon Task measuring response conflict monitoring processes. In contrast to the predominant amplitude modulations observed between Go and Nogo trials, the congruent modulations of the Simon paradigm are also characterized by latency shifts of the event-related potential components. Optimizing the cGAN architecture to grasp all functionally relevant aspects of electrophysiological signals is therefore an important future step.

Nevertheless, the results provide proof of principle that artificial intelligence methods (i.e., cGANs) may be useful to access neurophysiological principles and relationships between cognitive functions. They may be useful to derive and test computational principles underlying the interrelation of classes of goal-directed behavior on a neurophysiological level. The “parameters” which relate to these classes of goal-directed behavior and seem to be detected by the cGAN approach may be complex mathematical constructs that need to be estimated using artificial intelligence methods—at least when it comes to neurophysiological processes. This has substantial implications for cognitive science, in which processes underlying goal-directed behavior are increasingly seen and investigated as a dynamic interplay of control modes that are putatively regulated by a set of computational principles that have not yet been understood. At present, the research in this field is dominated by cognitive theory-driven approaches. In contrast, data-driven artificial intelligence methods like the ones used in this study have largely been neglected until now, possibly because deep networks are rarely built with biological plausibility in mind^16^. In this context, it will be a fundamental future challenge in the following steps to be taken to combine theory-driven (“why it works questions”) and data-driven approaches (“whether it works questions”)^16,35^ to derive a computational formalization underlying dynamic adjustments in goal-directed actions. We deem the current study to be a starting point for this in cognitive control and goal-directed behavior due to its conceptual motivations rooted in metacontrol theories on action control.

## Methods

### Participants

The study runs quantitative, within-subject manipulations of experimental conditions. A sample of *N* = 255 healthy student participants (121 females) took part in the study (convenience sample). The sample was gathered from different experiments. There were no data exclusions or participants’ dropouts. There was no randomization due to the within-subject design. The sample size is comparable to other studies applying deep learning to EEG data^18^. The mean age was 23.8 ± 2.8 years. All participants had a normal or corrected-to-normal vision and reported being free of any medication. During the recruitment phase, these participants reported not having any neurological or psychiatric disorder in a telephone interview. The participants received financial compensation or course credits for taking part in the study. The study was approved by the ethics committee of the University of Bochum (reference No. 3827-10/4490-12) and conducted in accordance with the Declaration of Helsinki. We obtained written informed consent from all study participants before any study procedure was started.

### Response inhibition task

To examine the inhibition of speeded responses, we used a Go/Nogo task in which the Go and Nogo conditions occurred with different frequencies (i.e., 70% Go trials and 30% Nogo trials). This ratio of Go and Nogo trials has been shown to increase demands on response inhibition processes because it encourages the execution of speeded responses^8–11^. Participants were seated approximately 60 cm from a 17-in. CRT screen. The experiment was presented using the software Presentation 14 (Neurobehavioral Systems, Inc.). As a Go stimulus, the word “DRÜCK” (German for “press”) was presented in white font on black background in the center of the screen for 200 ms. Participants were asked to respond by pressing a key with their right index fingers. As a Nogo stimulus, the word “STOPP” (German for “stop”) was presented in white font on a black background in the center of the screen for 200 ms. The trial ended as soon as the participant responded with a keypress (either a correct response in Go trials or a false alarm in Nogo trials) or 2200 ms after stimulus onset if no key was pressed (either a miss in Go trials or a correct inhibition in Nogo trials). The trials were separated by a jittered inter-trial interval (ITI) of 1000–1300 ms, in which only a central white fixation cross was presented on the screen. Overall, there were 315 Go and 135 Nogo trials. The entire experiment took about 15 min.

### EEG recording and processing

An EEG was continuously recorded during task performance with a sampling rate of 500 Hz using 64 sintered Ag/AgCl electrodes that were mounted in an elastic cap (EasyCap Inc.). EEG recording was performed using a QuickAmp amplifier (Brain Products Inc.), with the reference electrode placed at FCz. The signal was automatically re-referenced to a common average reference by the amplifier. The cap preparation was performed using Lectron III high chloride electrolyte gel to ensure that electrode impedances were kept below 5 kΩ. For offline data processing using the Brain Vision Analyzer 2 software package (BrainProducts Inc.), the data were down-sampled to 256 Hz. Then, a 0.5 Hz high-pass filter and an 18 Hz low-pass filter were applied (IIR, slope of 48 dB/oct). Subsequently, gross and irregular artifacts (e.g., technical artifacts, DC offset corrections, excessive movement/EMG artifacts, SQUID jumps) were discarded employing a manual raw data inspection procedure. This step was followed by an independent component analysis using the infomax algorithm to correct for horizontal and vertical eye movements, ECG, and pulse artifacts: Independent components showing these artifacts were discarded before the back-projection of the data to EEG sensor space was performed. After that, electrode FCz was interpolated using a spherical spline interpolation before the data was segmented into Go and Nogo trials using time markers in the EEG. Of note, only correct trials were segmented and included in all further data processing and analyses steps. Only Go trials with responses before the response deadline of 1200 ms and Nogo trials with no responses until the response deadline of 2200 ms were considered. The segments were initially 4000 ms long, starting 2000 ms before and ending 2000 ms after stimulus onset. An automated artifact rejection procedure followed segmentation to eliminate any artifacts that might have survived prior artifact removal steps. Segments were removed if any of the below-specified rejection criteria were met: a voltage step of more than 50 µV, a difference of values in a 200 ms intervals of >100 µV, amplitudes exceeding ±100 µV, a difference of minimum and maximum activity of less than 0.5 µV in intervals of 100 ms. This procedure removed on average 2.4 ± 3.6 (0.5%) of total trials. Then, a current source density transformation was performed (order of splines *m* = 4, maximum degree of the Legendre polynomials *n* = 10, precision of 2.72^−7^) to eliminate the reference potential. A baseline correction between −200 ms and stimulus onset was performed, and averages for Go and Nogo trials were formed on the single-subject level. On average, 242.4 ± 64.3 correct GO trials and 102.4 ± 26.8 correct NOGO trials remained for further analysis. A time window of 0 to 1000 ms relative to stimulus onset was used for the GAN procedures.

### Generative adversarial network (GAN)

GAN is a class of methods for learning generative models, which were initially proposed by Goodfellow^36,37^. GANs consist of two networks (i.e., a generator and a discriminator network) that compete in a game. While the generator tries to “fool” the discriminator by generating realistic/raw data from noise (i.e., *G*(*z*)), the discriminator takes a generated fake or real example as an input and makes a binary decision whether this input is fake (i.e., generated signal) or real (i.e., *D*(*x*)). Competition between these two networks results in improvements for both and in an ideal situation, the discriminator cannot distinguish real and fake data at the end of this process. This competition can be expressed as a zero-sum game with the following objective (i.e., *L*_Gan_)

1 $${L}_{{{{{\rm{Gan}}}}}}\left(D,G,x,z\right)=	\,{E}_{x\sim {P}_{{{{{\rm{data}}}}}}(x)}\left[{{{{\rm{log}}}}}\,\left(D\left(x;{\theta }_{D}\right)\right)\right]\\ 	+{E}_{z \sim {P}_{z}(z)}\,\left[{{{{{\rm{log}}}}}}\,\left(1-D\left(G\,\left(z;{\theta }_{G}\right);{\theta }_{D}\right)\right)\right]$$

2 $${{\theta}_{G}}^{*},{{\theta}_{D}}^{* }={{{{{\rm{arg }}}}}}\,{{\min }}_{{\theta }_{G}}{{\max }}_{{\theta }_{D}}{L}_{{{{{\rm{Gan}}}}}}(D,G,x,z)$$

**Fig. 5:**
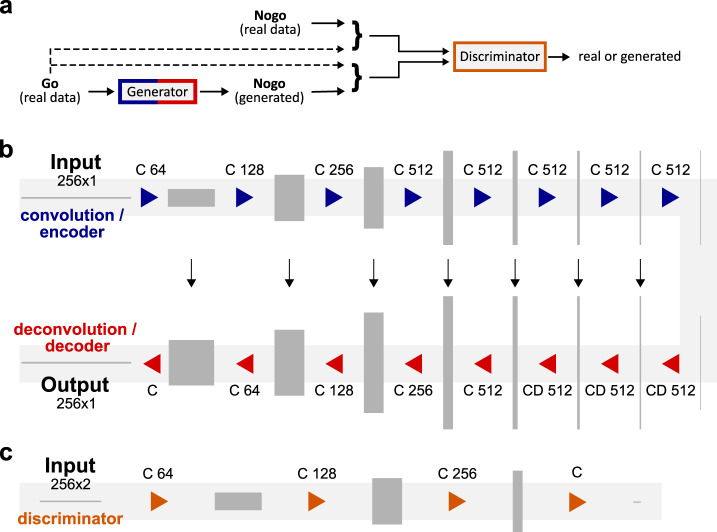
The general procedures and architectures of the conditional GAN for generating a Nogo signal based on the Go signal. **a** General procedures for generating a Nogo signal based on the Go signal. The real Go signal is fed into the generator. The real Go signal is concatenated with real and generated Nogo data separately. The discriminator assesses whether the pair of either real Go/real Nogo data or real Go/generated Nogo data is real or fake. **b** The architecture of the generator. It consists of an encoder (i.e., blue part) and a decoder (i.e., red part). Both decoder and encoder each consist of eight convolutional layers. Convolutions in the encoder down-sample by a factor of 2, whereas in the decoder, data is up-sampled by a factor of 2. Black arrows indicate a skip connection between each layer of the encoder and the decoder. **c** The architecture of the discriminator. It consists of four convolutional layers.

*D* and *G* are two neural networks with parameters *θ*_*D*_ and *θ*_*G*_ representing discriminator and generator, repetitively. *x* is an example of real data, and z is n-dimensional noise. ${P}_{{data}} \left(x \right)$ and ${P}_{z}\left(\right.z$) are the distribution of the data and noise, respectively. The objective of a GAN is to learn the mapping $z\mathop{\to }\limits^{G}x$ by estimating the parameters *θ*_*D*_ and *θ*_*G*_, in which the loss is maximal concerning *θ*_*D*_ and minimal concerning *θ*_*G*_. Goodfellow et al^37^. originally used simple neural networks (i.e., a multi-layer perceptron). After that, more complex architectures have been put forward (i.e., convolutional neural networks), which have been shown to lead to impressive results, especially in image generation^38–42^. Importantly, GANs can also be used as a tool for transforming a data distribution from one condition to another. This transformation process is called conditional GAN (cGAN). The principal architecture of a conditional GAN is shown in Fig. 5a.

Suppose that we have a dataset containing recorded data from two conditions (i.e., X and Y), which can be completely arbitrary. Conditional GANs are optimized to learn and estimate the function that transforms condition X to condition Y (e.g., transforming Go data into Nogo data). The procedure behind conditional GANs is very similar to that behind classical GANs. However, a slight difference between these methods lies in the construction of the generator. Classical GANs learn the mapping $z\mathop{\to }\limits^{G}x$ and the primary input is noise. In conditional GANs, however, the primary input is the real data from condition *x*. Using this information, the conditional GAN learns the mapping $x\mathop{\to }\limits^{G}y$^22^. In other words, the generator learns how to synthesize/ produce natural-looking data (i.e., y) by using *x* as its input. Conditional GANs have been widely used for the learning transformation of data such as image to image or text to image^22,43–45^. There are also a few studies that used GAN for the generation of EEG data. Two recent studies used GAN as an augmentation method to increase the dataset size^46,47^, and another study used a conditional GAN to generate EEG data similar to data recorded during epileptic seizures^48^. In the current study, we applied a conditional GAN^22^ to generate neurophysiological signals for the Nogo condition using data from the Go condition and vice versa. The chosen method by Isola et al^22^. is applicable when having a paired dataset. Supposedly, we have a dataset that recorded two conditions ${\left\{{x}_{i}\right\}}_{i=1}^{N}$ and ${\left\{{y}_{i}\right\}}_{i=1.}^{N}$ (i.e., Go and Nogo). When the correspondence between *x*_*i*_ and *y*_*i*_ exists for each example of the dataset, it is called a paired dataset. When there is no such correspondence, it is called an unpaired dataset. Our dataset was composed of averaged ERP data recorded during both Go and Nogo conditions for each subject. The number of examples is equal to the number of subjects and for each of them, there are both Go and Nogo conditions. Therefore, our dataset is paired. There are several methods for conditional GAN but it has been shown that the quality of results generated by the conditional GAN proposed by Isola et al^22^. is better (i.e., less blurred) than other methods in case the dataset is paired^45^. The loss function for the conditional GAN can be expressed as:

3 $${L}_{{{{{\rm{Gan}}}}}}\left(D,G,x,y\right)=	\,{E}_{x,y}\left[{\log }\,\left(D\,\left(x,y{{{{{\rm{;}}}}}}{\theta }_{D}\right)\right)\right]\,+{E}_{x}\,\left[{\log }\,\left(1-D\,\left(x,G\,\left(x{{{{{\rm{;}}}}}}{\theta }_{G}\right){{{{{\rm{;}}}}}}{\theta }_{D}\right)\right)\right]\\ 	+\lambda \,{E}_{x,y}\,\left[\,{\left|y-G\left(x\right)\right|}_{1}\right]\,$$

4 $${{\theta }_{G}}^{* },{{\theta }_{D}}^{* }={{{{{\rm{arg }}}}}}\,{{\min }}_{{\theta }_{G}}\,{{\max }}_{{\theta }_{D}}{L}_{{{{{\rm{Gan}}}}}}(D,G,x,z)$$

The first and second terms in Eq. 3 are very similar to classical GAN, but the input of the generator is real data *x* instead of noise, and the discriminator decides whether pair of (*x*,*y*) or (*x*,*G*(*x*)) is real or fake. The last term is a regularizer and prevents the generator from generating blurred images^49^. Transferred to the signal level, this means that the generator generates a signal containing high frequencies. Equation 3 states that the generator not only generates data that fools the discriminator but also considers that the *L*_1_ distance between generated and real data should be minimized. In other words, Eq. 3 can be considered as a normal autoencoder with *L*_1_ distance (i.e., last term in Eq. 3) plus adversarial loss (i.e., the first and second term in Eq. 3). Isola et al.^22^ used images as data for learning the transformation, but in our study, the data type was the time-resolved signal. Consequently, the architectures and parameters designed for images had to be changed to be applicable for time-series data. Specifically, we changed all of the convolutional and pooling layers from two dimensions into one dimension. Other hyper-parameters and network structures remained the same. Crucially, we employed *K*-fold as a cross-validation method to evaluate the transformation performance with *K* = 10. This means that 90% of subjects were randomly selected as a training set, and 10% were used for testing. By continuing this process ten times, all subjects in the dataset are part of both the testing and training sets for the conditional GAN. Please note that all of the results and plots are shown in the results section are based on the test sets in the *K*-fold method.

The architectures for the generator and the discriminator are shown in Fig. 5b, c^22^. The generator (Fig. 5b) consists of an encoder and a decoder block with a skip connection between each layer of the decoder and the encoder^50^. Assume that Ck denotes a Convolution-BatchNorm-ReLU layer with k filters and CDk denotes a Convolution-BatchNorm-Dropout-ReLU layer with a dropout rate of 50%. The decoder consists of 8 layers (i.e., C’64–C128–C256–C512–C512–C512–C512–C512). The encoder also has 8 layers in which layers 1–7 are CD512–CD512–CD512–CD512–C256–C128–C64, and the last layer is a convolution with a Tanh activation function to map to the output signal. The filter size for the convolution is 2 with a stride of 2. In the discriminator and the encoder, convolutions down-sample the data by factor 2, but in the decoder, they up-sample data by factor 2. A convolutional “PatchGAN” classifier was used as discriminator^22^. The discriminator tries to classify whether each of each patch in a signal is real or fake. The architecture of the discriminator is shown in Fig. 5c. The discriminator consists of five convolutional layers. The architecture for layers 1–4 is C’64–C128–C256–C512, and in the last layer, there is a convolution to map to one dimension output followed by a sigmoid activation function. The output shape of the discriminator is 16*1 (i.e., features), and the discriminator should assess whether each of the 16 output features (i.e., belongs to a patch of the input signal) is accurate or fake^22,51^. Please note that C’ 64, used in discriminator and encoder, is similar to C64, but there is no batch normalization. Furthermore, all ReLUs in the encoder and discriminator are leaky, with a slope of 0.2, while ReLUs in the decoder are not leaky. The conditional GAN was applied separately for each channel of the EEG data. The batch size, number of the epochs, and *λ* are set to 1, 15, and 100, respectively. Finally, please note that we do not aim to design a model that can achieve the highest performance for the transformation of EEG data in two different conditions in this study. Our primary goal is to use a model with acceptable performance to transform EEG data from automated behavior to inhibitory behavior. In other words, we want to show that instances of goal direct behavior (i.e., Go and Nogo) have an interrelation at the neurophysiological level. Thus, we used a well-established deep learning architecture^22^ able to transform images from one condition to another. The model’s performance was already validated in various experiments^22^ such as semantic data labels↔photo, architectural labels→photo, map↔aerial photo, edges→photo, etc., with identical hyperparameters and network architecture as used in the current study. Thus, the only modification that we applied is changing all convolution and pooling layers from two dimensions into one dimension applicable to the EEG signal. To increase the signal-to-noise ratio in the data used for learning, we did not use single-trial data but used the single-subject averages of data in the respective conditions. The cGAN analysis was run using a Jupyter Notebook (Project Jupyter) and Python 3.8.5 (Python Software Foundation).

### Behavioral and standard ERP data analysis

For descriptive data, the mean and standard error of the mean is given. For the quantification of the ERP components, the mean activity in an individually determined time window was used. The electrodes were chosen according to their respective scalp topography. Electrodes P7/P9 and P8/P10 were used for the P1 and N1 components. The P1 was quantified as the mean activity in the time window from 90 to 110 ms after stimulus onset. For the N1 component, the time window of 150–170 ms was chosen. The N2 was quantified in the time window of 250–280 at electrode Cz. The P3 was quantified in the time window from 375 to 400 ms at electrode FC1 (frontocentral P3) and at electrode P1 (parietal P3). The ERP data standard analysis was performed using repeated-measures ANOVA. The model included the within-subject factors “condition” (Go vs. Nogo trials) and “electrode,” whenever applicable. Post-hoc tests were Bonferroni-corrected.

### Comparison of real and generated EEG data

For comparison of the real (recorded) Nogo data (Nogo_real_) and the generated Nogo data from the GAN (Nogo_generated_), cluster-based permutation tests were computed using field trip toolbox^52^. Real and generated data for every single channel were compared from 0 ms to 1000 ms using dependent t-tests corrected for multiple comparisons using the cluster method (1000 random draws, alpha = 0.05, minimum number of neighborhood channels required for a selected sample = 2). Consecutively, Bayesian *t*-tests were conducted to compare each single data point from 0 to 1000 ms for the pooled activity of electrodes as revealed by the preceding cluster-based permutation tests.

### Source localization (sLORETA) of real and generated EEG data

To examine the similarities between the original and the generated Nogo signal at the source (functional neuroanatomical) level, we conducted source localization using sLORETA^53^. We contrasted the following conditions for this analysis: Nogo_real_ > Go_real_ and Nogo_generated_ > Go_real_ and show the results in the sLORETA-provided MNI-brain template www.unizh.ch/keyinst/NewLORETA/sLORETA/sLORETA.htm. For source reconstruction, the sLORETA method uses a three-shell spherical head model. The intra-cerebral volume is partitioned into 6239 voxels within this head model using a spatial resolution of 5 mm. Then, the standardized current density is calculated for every voxel using an MNI152 head model template. Several studies have corroborated the reliability of source estimations provided by sLORETA^54–57^. To correct for multiple comparisons in the contrasts, we used the sLORETA built-in voxel-wise randomization tests with 2000 permutations. This procedure is based on statistical non-parametric mapping procedures. Locations of significantly different voxels (*p* < 0.05, two-sided) are shown in the MNI-brain. The colors show critical *t*-values in the MNI brain.

### Statistics and reproducibility

The sample size is comparable to previous studies using deep learning on EEG data^18^. Details about the *K*-fold cross-validation of the cGAN results are given in Section “Generative adversarial network (GAN)”. Details about behavioral and ERP data analysis in Section “Behavioral and standard ERP data analysis”. All information about the statistical comparison of generated and real data can be found in Section “Comparison of real and generated EEG data”.

### Reporting summary

Further information on research design is available in the Nature Research Reporting Summary linked to this article.

## Supplementary information

Supplementary Information

Reporting Summary

## Data Availability

Data can be downloaded from https://osf.io/6n7uc. All other data are available from the corresponding author upon reasonable request.

## Code availability

The GAN architecture can be downloaded from https://phillipi.github.io/pix2pix/. Further custom code used to process the data can be found here https://osf.io/6n7uc. The code to run the cGAN has been deposited in OSF and ZENODO^58^.
