# Supplementary material for: Conditional generative adversarial networks applied to EEG data can inform about the inter-relation of antagonistic behaviors on a neural level
Source: Commun Biol. 2022 Feb 21;5:148. doi: 10.1038/s42003-022-03091-8 (PMC8861069; doi:10.1038/s42003-022-03091-8)
Supplement: Supplementary file 1 — Supplementary Information [file 42003_2022_3091_MOESM1_ESM.pdf]

Supplemental material

Conditional Generative Adversarial Networks applied to EEG data can inform about the inter-relation of antagonistic behaviors on a neural level

Amirali Vahid[1], Moritz Mückschel[1], Sebastian Stober[2], Ann-Kathrin Stock[1], Christian Beste[1]

1 Cognitive Neurophysiology, Department of Child and Adolescent Psychiatry, Faculty of Medicine, TU Dresden, Germany
2 Artificial Intelligence Lab, Institute for Intelligent Cooperating Systems, Faculty of Computer Science, Otto von Guericke University Magdeburg, Germany

Simon paradigm cGAN

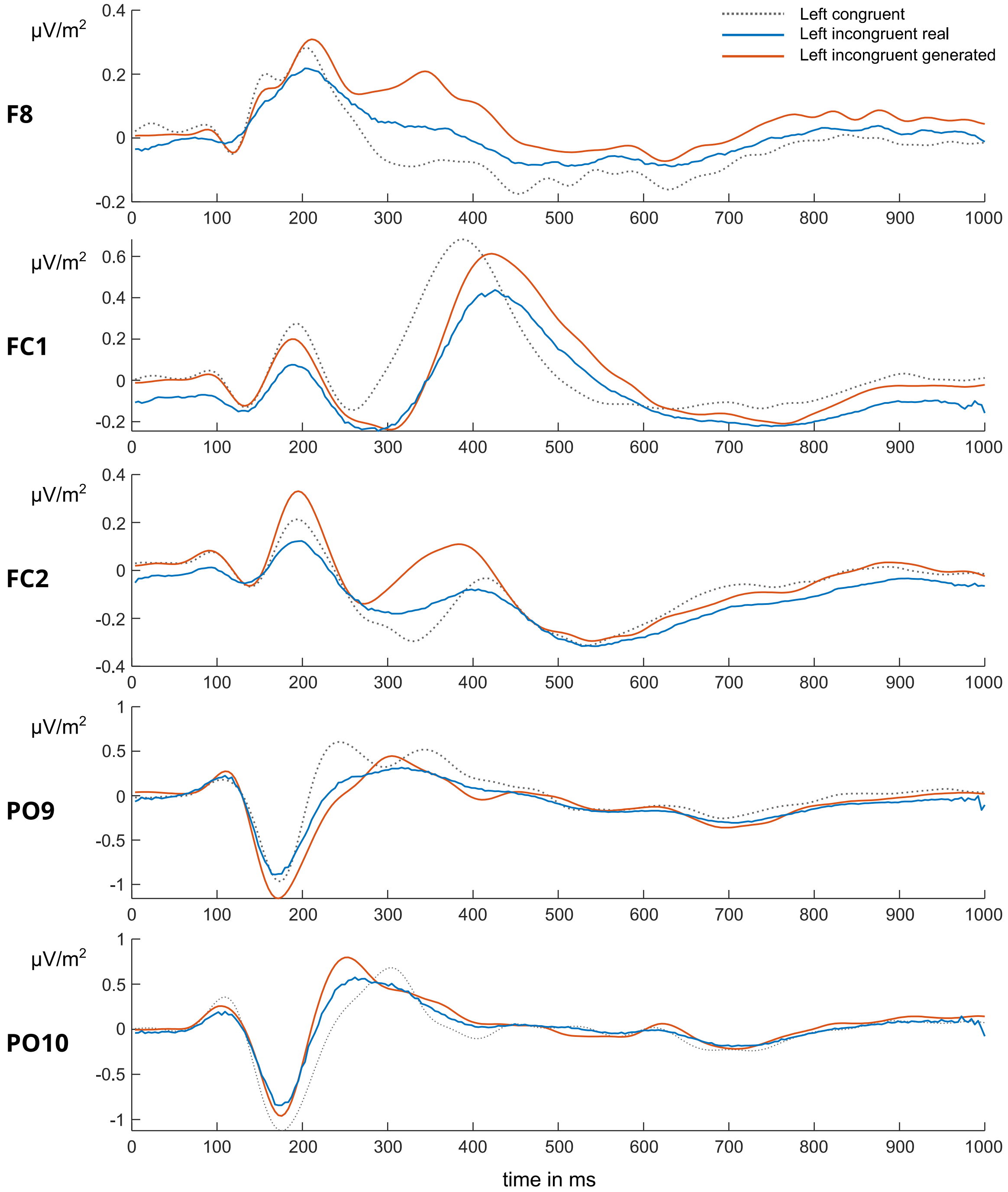

Supplemental Figure 1: Results of cGAN applied on Simon paradigm data.

We applied the same cGAN architecture on a Simon task paradigm data published by Vahid et al. (2020) [1], a sample consisting of N=186 healthy adult participants. The Simon task measures response conflict monitoring processes and therefore another central aspect of cognitive control [2]. The input of the model was the data of left hand response congruent trials, the output was incongruent left hand response. The cGAN was applied on data from channels F8, FC1, FC2, PO9 and PO10. Vahid et al. identified these channels as being vital for deep learning to classify the trial type in this dataset [1]. As can be seen in Figure S1, the generated incongruent signal (orange line color) considerably differs from the real incongruent signal (blue line color). Deviations can be seen both on the level of the signal amplitudes as well as on a temporal level i.e., peak latencies.

Supplementary References

1. Vahid, A., Mückschel, M., Stober, S., Stock, A.-K. & Beste, C. Applying deep learning to single-trial EEG data provides evidence for complementary theories on action control. *Commun Biol* **3**, (2020).
2. Keye, D., Wilhelm, O., Oberauer, K. & Stürmer, B. Individual differences in response conflict adaptations. *Front Psychol* **4**, 947 (2013).
